# Spleen and head kidney differential gene expression patterns in trout infected with *Lactococcus garvieae* correlate with spleen granulomas

**DOI:** 10.1186/s13567-019-0649-8

**Published:** 2019-05-02

**Authors:** Rosario Castro, Julio Coll, María del Mar Blanco, Antonio Rodriguez-Bertos, Luc Jouneau, José Francisco Fernández-Garayzábal, Alicia Gibello

**Affiliations:** 1 Department of Animal Health, Faculty of Veterinary Sciences, Complutense University, Madrid, Spain; 2 INRA, Virologie et Immunologie Moléculaires, Université Paris-Saclay, Jouy-en-Josas, France; 3 Department of Biotechnology, Instituto Nacional Investigaciones Agrarias y Alimentarias, INIA, Madrid, Spain; 4 Department of Internal Medicine and Animal Surgery, Faculty of Veterinary Sciences, Complutense University, Madrid, Spain; 5 VISAVET Animal Health Surveillance Center, Complutense University, Madrid, Spain

## Abstract

*Lactococcus garvieae* is a significant pathogen in aquaculture with a potential zoonotic risk. To begin to characterize the late immune response of trout to lactococcosis, we selected infected individuals showing clinical signs of lactococcosis. At the time lactococcosis clinical signs appeared, infection by *L. garvieae* induced a robust inflammatory response in the spleen of rainbow trout, which correlated with abundant granulomatous lesions. The response in kidney goes in parallel with that of spleen, and most of the gene regulations are similar in both organs. A correlation existed between the early inflammatory granulomas in spleen (containing macrophages with internalized *L. garvieae*) and up-regulated gene sets, which defined the presence of macrophages and neutrophils. This is the first analysis of the immune transcriptome of rainbow trout following *L. garvieae* infection during the initiation of adaptive immune mechanisms and shows a transcriptome induction of antibody response by both IgM (+) and IgT (+) spleen B cells to respond to systemic infection. These results increase our understanding of lactococcosis and pave the way for future research to improve control measures of lactococcosis on fish farms.

**Electronic supplementary material:**

The online version of this article (10.1186/s13567-019-0649-8) contains supplementary material, which is available to authorized users.

## Introduction

Fish lactococcosis is a haemorrhagic septicaemia caused by *Lactococcus garvieae.* This pathogen has been isolated worldwide from numerous cultured and wild fish species, but lactococcosis is particularly prevalent and economically relevant in farmed rainbow trout (*Oncorhynchus mykiss*), and usually occurs when water temperatures rise to 18 °C [1, 2]. Despite its major relevance as a fish pathogen, it has also been isolated from clinical specimens in cows and water buffalos with subclinical mastitis and pigs with pneumonia [3, 4]. Moreover, *L. garvieae* has been recently involved in human infections and is considered an emerging opportunistic and potentially zoonotic pathogen [5, 6]. Vaccination is the best measure to prevent fish lactococcosis [1, 2]. However, commercially available vaccines are not fully effective for all fish species nor do they protect fish for extended periods, and lactococcosis outbreaks sometimes occur in vaccinated fish [7, 8]. To improve vaccines, a better knowledge of the immune response to *L. garvieae* infection of fish is desirable.

Analysis of fish immune responses to pathogenic bacterial infections has benefited from the application of transcriptome profiling technologies. Thus, microarrays have been used to study the transcriptomic responses following exposure to different bacterial fish pathogens such as *Streptococcus iniae* [9] and *Aeromonas salmonicida* [10]. For *L. garvieae*, the early transcriptome response of immune organs following a lactococcosis-induced infection in grey mullet (*Mugil cephalus*) has been recently reported [11]. However, similar transcriptome studies analysing the immune response to *L. garvieae* infection in rainbow trout are missing, despite that this fish species is the most affected by *L. garvieae* infections [1, 2]. Therefore, in the present study, we performed a transcriptome analysis of the spleen and head kidney of rainbow trout experimentally infected with *L. garvieae* compared with non-inoculated individuals. Moreover, most studies investigating the immune transcriptome have used microarrays designed from expressed sequence tags (EST) derived from whole genomes [12]. A drawback of this approach is that transcripts of many immune-related genes are often under-represented. For this reason, in this work we have used a custom designed immune-targeted microarray specifically designed to contain a higher number of transcripts derived from immune-related mRNAs deposited in gene or pathway data banks [13]. This microarray has been successfully used to study the immune response in rainbow trout [13–16].

We compared different immune response transcriptome profiles between the spleens and head kidneys of infected trout, which correlated with the appearance of abundant granulomas in the spleen. The results of this study provide insights into the innate and early adaptive immune response mechanisms that are activated after lactococcosis infection in rainbow trout. These insights could help to develop more efficient strategies for controlling lactococcosis in fish aquaculture.

## Materials and methods

### Bacterial and fish sources

*Lactococcus garvieae* 8831 (Lg8831), a clinical strain isolated from diseased rainbow trout affected by lactococcosis and representative of most natural outbreaks in Spain [17] was used for the experimental infections. Lg8831 was grown aerobically in BHI broth (BioMérieux, Marcy l’Etoile, France) at 29 °C and harvested at the mid-log phase (OD_600_ ~ 0.9). For the experimental infection, healthy rainbow trout (*Oncorhynchus mykiss*) of approximately 10 g and 11–13 cm were obtained from a lactococcosis-free fish farm in which the animals had not been vaccinated against *L. garvieae*. Five individuals were randomly sampled to certify absence of *L. garvieae* by PCR [18]. The trout were randomly divided into two groups (challenge and control), which were maintained in two separate continuously aerated tanks, for an acclimation period of 1 week at 14 °C and were fed twice daily with commercial pellets.

### Experimental infection procedure

Rainbow trout were anaesthetized with 100 mg L^−1^ of tricaine methane sulfonate (MS-222) for 5 min. The challenge group (*n* = 20) was intraperitoneally inoculated with 100 µL of 2.65 × 10^3^ Lg8831 cells diluted in phosphate buffered saline (PBS) since 50% of the lethal dose of this strain was established as < 10^4^ CFU/fish (data not shown). The bacterial concentration of the inoculum was determined by ten-fold serial dilutions and further plated onto Columbia 5% sheep blood agar plates (CNA, BioMérieux). Trout of the non-inoculated control group (*n* = 10) were inoculated with 100 µL of PBS.

After the inoculation, the water temperature in the tanks (including the control group) increased gradually from 14 to 18 °C. Fish were observed twice daily for 5 days post-inoculation, and mortality was recorded. Dead fish were analysed microbiologically to confirm that the mortality was due to *L. garvieae* infection. Samples of liver and head kidney were cultured on CNA plates (BioMérieux) and incubated for 72 h at 30 °C. The grown bacteria were identified using the API Rapid ID32 Strep system (BioMérieux) and by PCR [18].

### Sampling of organs for microarray and histopathological studies

Most infected trout started to exhibit clear clinical signs of lactococcosis at 72 h post-inoculation, including exophthalmia, splenomegaly, haemorrhagic liver and intestine, and inflamed kidney. The spleen and head kidney from six sick fish and four control fish taken at 72 h post-inoculation were used for further analysis. Half of each organ was stored in RNA later solution (Ambion, Thermo Fischer Scientific) at −80 °C until RNA extraction for further microarray experiments, and the other half was used for histopathological examination. No clinical signs or mortalities were registered in fish of the control group.

### Histopathological and immunohistochemical examination

For histopathological analysis, the spleen and kidney samples of the same infected trout used for microarray experiments were fixed with 10% buffered formalin (Panreac A3684), stabilized with methanol at pH 7 for 24 h at room temperature, embedded in paraffin, cut into 4 µm slices and stained with haematoxylin and eosin.

The immunohistochemical study was carried out in the same tissues used for histological analysis by a streptavidin–biotin–peroxidase complex method, using a polyclonal rabbit antibody serum against Lg8831 as previously described [19], on Novolink Polymer Detection Systems Novocastra (Leica RE 7280-CE). We have included a 10% hydrogen peroxide incubation for 1 h during the endogenous peroxidase blocking step in order to reduce the melanin pigment.

### Microarray analysis

#### RNA extraction and purification

Total RNA from spleen and head kidney samples in RNA later was isolated from control and inoculated trout. RNA obtained after TRIzol reagent (Invitrogen) extraction was treated with DNase on column using the RNeasy Plus kit (Qiagen). The concentration was estimated on a NanoDrop 1000 spectrophotometer (NanoDrop Technologies, Inc., Rockland, DE, USA). Individual RNA samples were pooled into 3 groups (2 fish per pool) for control and *L. garvieae*-infected conditions for each organ. The quality and concentration of RNA was determined using the RNA 6000 NanoKit on the Bioanalyzer 2100 (Agilent Technologies, Palo Alto, CA, USA) at the Genomics Unit facilities (Parque Científico de Madrid). Only high-quality RNA samples (RNA integrity number or RIN > 9) were used.

#### Microarray experimental design and hybridization of trout transcripts

We used a rainbow trout microarray (8 × 15 K) called Minitrout 12.8 (Agilent ID032303) that was previously designed and validated [13–16]. This immune-targeted microarray contains 6442 unique 60-mer oligo sequences, each in duplicate and arranged randomly [13, 14]. Rainbow trout genes were classified as belonging to immune pathways according to the Kyoto Encyclopedia of Genes and Genomes (KEGG) and the WikiPathways (WIKI) databases as previously described [16]. Rainbow trout genes to derive each of the probes were also manually selected as previously described [13–16]. To first study the transcriptional responses, the corresponding probes were classified according to different gene groups [13]: AM, antimicrobial peptides (number of probes, *n* = 49); C, complement components (*n* = 176); CD, cluster differentiation antigens (*n* = 58); CK, chemokines and their receptors (*n* = 121); HSP, heat shock proteins (*n* = 247); IFN, interferons and their receptors (*n* = 91); IL, interleukins and their receptors (*n* = 119); MA, macrophage related genes (*n* = 125); MX, interferon-inducible proteins (*n* = 7); TLR, toll-like receptors (*n* = 31); TNF, tumour necrosis factor (*n* = 32); TR, transcription factors (*n* = 671); and VIG, VHS virus-induced genes (*n* = 26). Three groups of multivariable gene families were also included: MHC, major histocompatibility complex (*n* = 320); IG, immunoglobulins (*n* = 914) and TCR, T cell receptors (*n* = 120) [13].

Labelling of RNA from the trout organ samples and microarray hybridizations was performed by the NimGenetics Company (Madrid, Spain) in compliance with the Minimum Information About a Microarray Experiment (MIAME) standards. Four 8 × 15 K slides were used in this study, giving a final distribution of samples as follows: 3 pools of spleen (MB04-MB06) and head kidney (MB09-MB011) samples of *L. garvieae*-inoculated trout, 3 pools of spleen (MB01-MB03) and 2 pools of head kidney (MB07 and MB08) samples of control trout. Three replicates of all pooled samples (except two replicates for control sample MB07) were performed (Additional file 1).

#### Microarray data analysis

Micro-array signal was normalized using intra-array median subtraction after log_2_ transformation.

Differential analysis was performed using limma R package. Raw and normalized data were deposited into the GEO database (GSE101695). Differential expression analysis between infected samples and control samples was performed for each tissue. *p* values were corrected for multiple testing using Benjamini–Hochberg procedure. Adjusted *p* values lower than 1% with an absolute log fold change of expression greater than 1 were considered for subsequent analyses (or the corresponding ratio > 2 and < 0.5).

Gene set enrichment analysis (GSEA) [20, 21] was used to analyse the data of different gene sets (GS). Briefly, the list of all gene hybridization values was ranked using the GSEA t-test statistic metric obtained by comparing the phenotypes from *L. garvieae*-infected trout versus the control. GS from different immune-related pathways classified according to GSEA enrichment scores (ES) were used and compared to each other using normalized ES (NES), which corrects for the number of GS genes. For assessing NES significance, the false discovery rate (FDR) was used after comparing with the corresponding null distribution obtained after averaging 1000 GS permutations (1000 random gene combinations per GS). As suggested by GSEA, the most stringent cut-off value of FDR q < 0.05 was used for NES significance.

### Reverse-transcription quantitative real-time PCR (RT-qPCR)

The Agilent ID032303 array was already validated in previous studies on the trout transcriptome in response to vaccination, viral proteins and thyroid-active compounds [13–16]. We performed real-time RT-qPCR analysis of selected genes to double-check the microarray results. The RT-qPCR primers were obtained from existing literature [22, 23], using the elongation factor 1 alpha (EF-1α) as house-keeping gene (Additional file 2). The cDNA was synthesized from the RNA samples used for the microarray hybridization, following the Revert Aid First Strand cDNA Synthesis Kit (Thermo Fischer Scientific) protocols. Amplification was performed in a CFX96 Real-Time PCR Detection System (BioRad) in triplicate for each sample using Power SYBR Green PCR Master Mix (Thermo Fischer Scientific). The PCR amplification was initiated at 95 °C for 10 min followed by 40 cycles of 95 °C for 15 s and 60 °C for 1 min. The threshold cycle value (Ct) was obtained by the automatic position of the threshold baseline at the mid-exponential phase of the curve. Data normalization and analysis were performed using CFX Manager Software Vs 3.1.

## Results

### Histopathological analysis of spleen and head kidney

**Figure 1:**
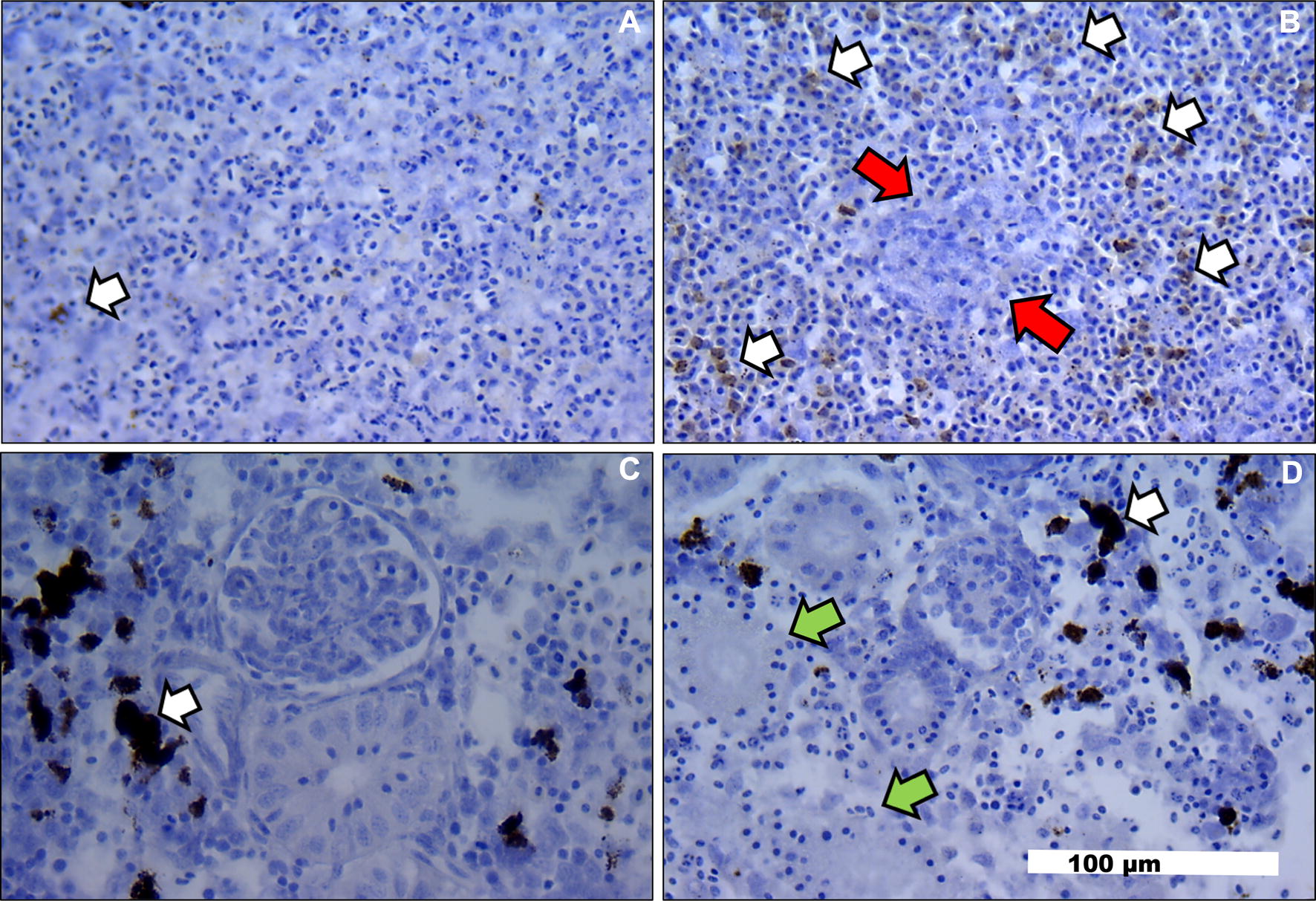
**Histopathological examination of spleen and head kidney from** ***L. garvieae*-infected and uninfected rainbow trout. A** Spleen from uninfected trout. **B** Spleen from *L. garvieae*-infected trout. **C** Head kidney from uninfected trout. **D** Head kidney from *L. garvieae*-infected trout. White arrows, melanomacrophage groups in spleen and head kidney. Red arrows, granulomatous lesion of spleen. Green arrows, moderate tubular necrosis in head kidney. Horizontal bar, 100 µm.

**Figure 2:**
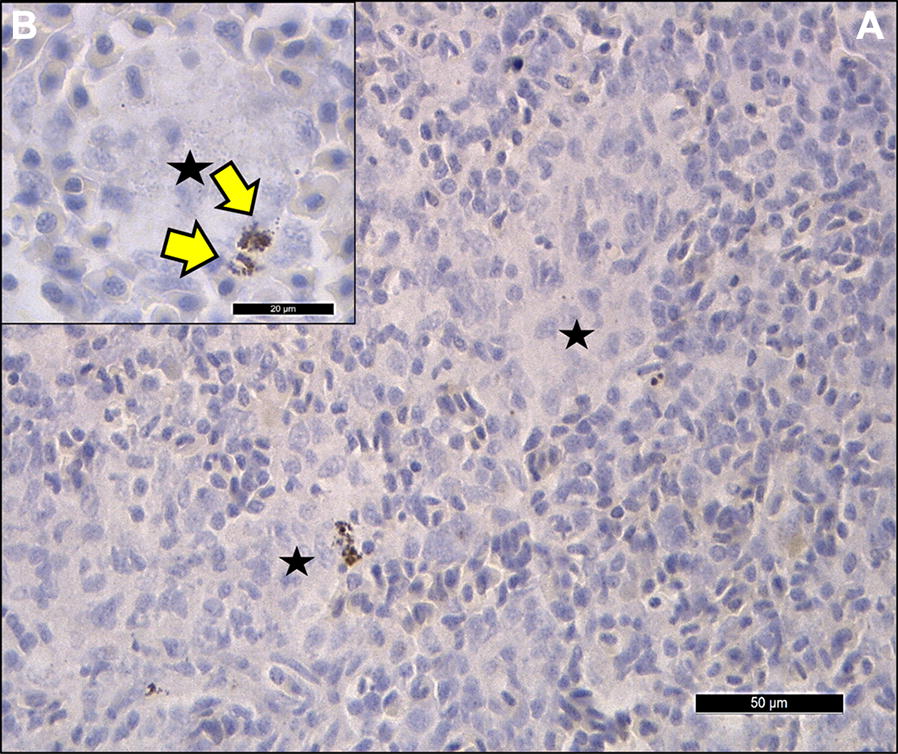
**Immunohistochemical examination showing a granulomatous lesion of spleen from** ***L. garvieae*-infected rainbow trout.** The immunohistochemical staining was performed with a polyclonal antibody serum against *L. garvieae.* **A** Spleen from *L. garvieae*-infected trout showing early macrophage granulomas (black stars); Horizontal bar, 50 μm. **B** Insert from splenic granuloma (black star) showing infiltrated macrophages with internalized *L. garvieae* (yellow arrows); horizontal bar, 20 μm.

Spleens obtained from *L. garvieae*-infected trout showed an increased number of melanomacrophages in the red pulp (Figure 1B, white arrows) compared to that in the uninfected control trout (Figure 1A). Early macrophage granulomas were also observed in the spleen of *L. garvieae*-infected trout (Figure 1B, red arrows). By contrast, no increase in melanomacrophage numbers and a moderate tubular necrosis were observed in the head kidney of *L. garvieae*-infected trout (Figure 1D, green arrows) compared to those in the uninfected control trout (Figure 1C). The presence of *L. garvieae* in macrophagic cells (Figure 2B, yellow arrows) into granulomatous lesions of the spleen red pulp (Figure 2A) was confirmed by immunohistochemical staining, confirming the intense inflammatory response in this tissue.

### Overview of spleen and head kidney immune-related transcripts from *L. garvieae* infected trout

**Figure 3:**
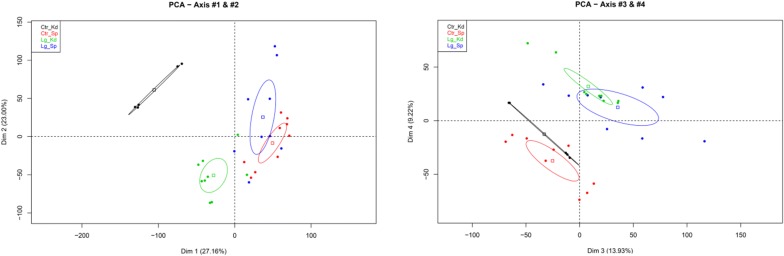
**Global analysis of microarray data.** Principal component analysis of the transcriptomes of spleen and head kidney samples from control and *L. garvieae*-infected rainbow trout. Projection on the four first axis is shown (left panel-dimension 1: horizontal axis, dimension 2: vertical axis; right panel-dimension 3: horizontal axis, dimension 4: vertical axis).

To increase the detection of immune-related transcripts expressed at low concentrations after *L. garvieae* infection, an immune-targeted microarray designed with selected genes from GenBank mRNA published sequences was used. Figure 3 shows the principal component analysis (PCA) performed to give an overview of the global responses. The transcriptomes were separated primarily by tissues as seen with dimension 1 (Dim 1 horizontal axis, Figure 3 left), with spleen datasets closely grouped but different from *L. garvieae* head kidney datasets and even more distant from control head kidney. Dim 2 (vertical axis) seems to represent the intragroup variation, with the highest variation observed among the *L. garvieae* spleen transcriptomes analysed. A clear discrimination was obtained between the control and the *L. garvieae* infected datasets as is shown in Dim 3 (horizontal axis, Figure 3 right) and Dim 4 (vertical axis).

Globally, transcripts for membrane and secreted heavy chain of IgM, complement component *c6*, several cathepsins, *beta*-*2 microglobulin* and heat shock proteins were the strongest up-regulated probes in spleen and head kidney (Additional file 3, Top50 up- and down-regulated probes). Among spleen top50 up-regulated probes also appear *interferon inducible protein Gig2*, *lmp2*, *cystatin b*, *galectin*, *chitinase*, *vcam* and *perforin*, whereas in head kidney top50 up-regulated outlie *alpha tubulin*, *beta*-*thymosin* and *ubiquitin*. Strongest down-regulated probes in both organs include complement components *c3* and *bf2*, several transcription factors, *ck11*, *small inducible cytokine b14*, *il1b*, *cxcr3a*, and *ccr5*. Differential down-regulated probes include several beta-defensins, *il17d*, *il20*, *il20 precursor*, *ck8a* and complement factor *H1* in spleen; *leap2a*, *il18*, *cxcd1*, *stabilin* and *ifng1* in head kidney.

### Transcript profile analysis of different immune cells studied by cellular-specific gene sets (cGS) and RTqPCR

Because anti-protein reagents to detect markers of trout immune cells were unavailable, we used a GS list of genes (cellular specific gene sets, cGS) related to TH1, TH2, TH17 (T helper cells), CTL (cytotoxic T cells), TREG (T regulatory cells), B (IgM expressing B cells) and BT cells (IgT expressing B cells), dendritic cells, NK cells (natural killer), macrophages, and neutrophil cells to discriminate among different immune-related cellular subsets [13–16]. NK cells, CTL and TREG were up-regulated in the head kidney, while macrophages and neutrophils were up-regulated in the spleen (Table 1).

**Table 1:** **Comparison of normalized enrichment scores (NES) obtained by using gene set enrichment analysis (GSEA) of cellular gene-sets (cGS)**

cGS	No. genes per cGS	Spleen	Kidney
NKCELLS	20	1.05	1.72*
CTLs	9	0.93	1.60*
TREG	11	1.15	1.42*
TH2	14	1.19	1.32
MACROPHAGES	16	1.62*	1.29
TH1	13	1.33	1.24
TH17	18	1.02	0.74
B	9	0.92	−0.52
BT	8	0.93	−0.53
NEUTROPHIL	7	1.44*	−0.80
DENDRITIC	5	−0.94	−1.27

The Table shows the NES of each cGS on the different trout phenotypes ordered by those of kidney.

NK-cells: natural killer cells, CTL: antigen-specific cytotoxic T cells, Treg: T regulatory cells, Th2: T-helper 2 cells, Macrophages: monocyte/macrophages, Th1: T-helper 1 cells, Th17: T-helper 17 cells, B cells: IgM producing cells, BT cells: IgT producing cells, neutrophil: neutrophil and granulocyte cells, dendritic: dendritic cells.

* FDR q value < 0.25.

**Figure 4:**
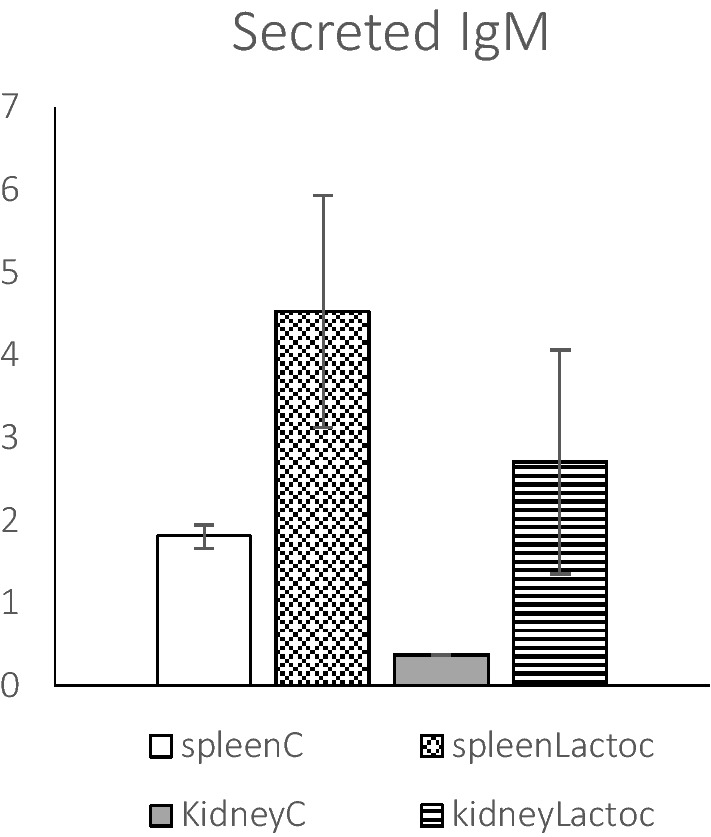
**RT-qPCR relative gene expression values of secreted IgM.** The relative gene expression values were determined by RT-qPCR in spleen and kidney as the mean of expression relative to the endogenous control *ef1a.* Means and standard deviations (*n* = 3), were calculated by the formula, expression value of each gene/expression value of *ef1a*. The *p* values are shown as *p < 0.05.

The gene probes corresponding to heavy and light chain membrane-bound form of IgM and IgD (defining B cells) were up-regulated in the spleen (Table 1), whereas the membrane form of IgT (defining BT cells) was only lightly up-regulated in this tissue. Probes for secretory forms of IgM were highly up-regulated in the spleen and head kidney of *L. garvieae*-infected trout. The up-regulation of secreted IgM transcripts in these tissues of *L. garvieae*-infected trout was confirmed by RT-qPCR (Figure 4). A positive correlation (r = 0.952) was observed between the microarray and RT-qPCR data (Additional file 4).

Among the genes related with antigen presentation, multiple probes of MHC class I antigens, b-2 microglobulin and tapasin appeared up-regulated in both organs of *L. garvieae*-infected fish, as well as *nitr2* (novel immune type receptor 2), *pigr* (polymeric immunoglobulin receptor), and *tcra*, *tcrb* (T cell receptor alpha and beta chain, constant region). *lck1* and *lck2* (lymphocyte-specific protein tyrosine kinase, involved in lymphocyte intracellular signaling pathways) appeared up-regulated in the spleen, and *nitr4* appeared down-regulated in both organs.

### Gene set enrichment analysis (GSEA) of immune-targeted microarray data

Table 2 shows the pathways that were regulated in the spleen compared to those in the head kidney after *L. garvieae* infection. Among these pathways, the NOD and complement pathways were up-regulated in spleen of *L. garvieae*-infected trout. The NOD-like receptor signalling pathway, according to the KEGG database, is known to be activated by bacterial peptidoglycans, among other ligands, resulting in activation of the NF-kappa B (NF-KB), MAPK, IL6 and IL8 signaling pathways in mammals. These theoretical final activations were observed by up-regulation of the MAPK signaling pathway detected in the spleen of *L. garvieae* infected trout (Table 2) together with *p100/p52 (nfkb)* and IL-8 genes. On the other hand, the fibroblast growth factor signaling pathway was down-regulated in the spleen. In the head kidney, the apoptosis pathway and NF-KB signaling pathway were significantly up-regulated.

**Table 2:** **Comparison of normalized enrichment scores (NES) obtained by using gene set enrichment analysis (GSEA) of immune-related pathways**

Pathway	Size	Spleen	Kidney
		NES	NES
COMPLEMENT-K	12	1.66*	1.05
NOD-K	12	1.45*	1.31
MAPK-K	17	1.43*	−0.84
APOPTOSIS-K	12	1.41	1.52*
NFKB-K	26	1.26	1.50*
FGF SIGNALING PATHWAY-W	7	−1.64*	−1.06

The Table shows the NES values of each GS ordered by those of spleen.

Terminal-K: KEGG pathway, Terminal-W: Wikipathways, NOD: nucleotide-binding oligomerization domain-like receptors, MAPK: mitogen-activated protein kinase, NFKB: nuclear factor k-light-chain-enhancer of activated B cells, FGF: the fibroblast growth factor signalling pathway.

* Italic, FDR q value < 0.25. Size: number of genes analysed.

### Regulation of selected gene groups from the microarray data

#### Cluster differentiation antigens (CDs) and macrophage related genes

**Figure 5:**
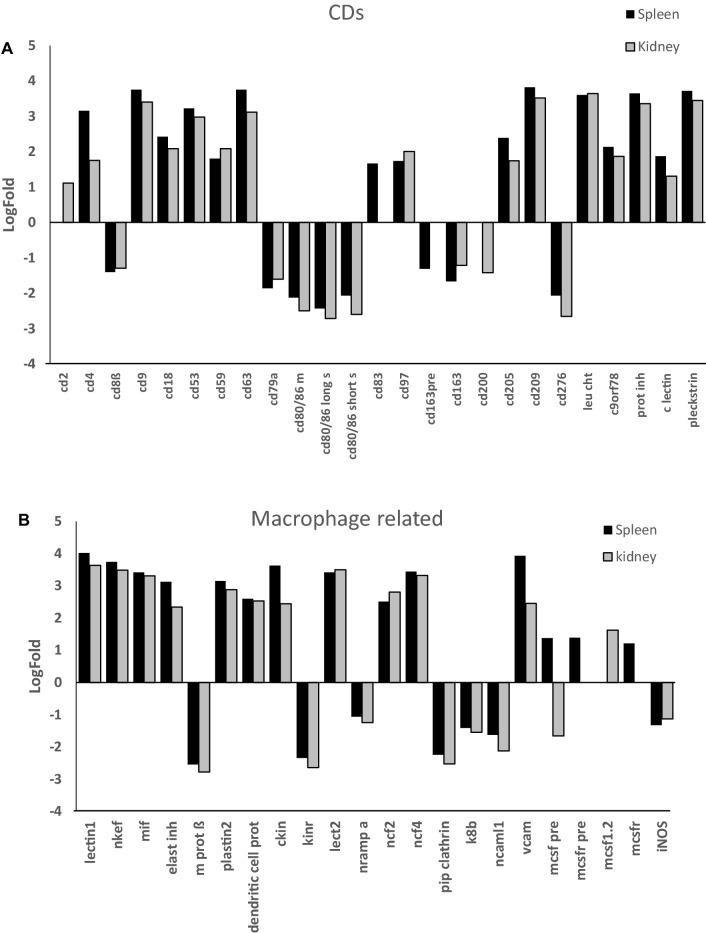
**Differential transcript expression of cluster differentiation antigens (A) and macrophage related genes (B) in** ***L. garvieae*-infected trout.** Trout were injected with *L. garvieae* and RNA was extracted from spleen and head kidney of symptomatic (*n* = 6) and of control (*n* = 6) fish for microarray hybridization. After normalization, the mean and standard deviations were represented as differential expression LogFold from up- (+) or down- (−) regulated transcripts (> 1 or < −1, respectively), calculated by the formula, normalized fluorescence of each gene in *L. garvieae*-infected trout/normalized fluorescence of each gene in uninfected trout.

Among the cluster differentiation antigens (CDs) analysed, transcripts up-regulated in spleen and head kidney of *L. garvieae* infected fish were *cd3* (T cell marker), *cd4* (T-helper cell marker), *cd9* (up-regulated in activated lymphocytes), *cd18* (leucocyte adhesion molecules), *cd53* (related to T cell and NK activity), *cd59* (involved in complement-mediated cell lysis and T cell activation), *cd63* (platelet activation marker), *cd97* (related to leucocyte adhesion, recruitment and activation), *cd205* (involved in antigen uptake, processing and presentation by dendritic cells), *cd209* (C-type lectin receptor of macrophages and dendritic cells, which may increase phagocytosis), *leu cht* (leucocyte chemotaxin), *c9orf78* (involved in gene splicing), *prot inh* (proteinase inhibitor), *pleckstrin* (platelets) and a *C*-*type lectin receptor b* (Figure 5A). *cd83* (involved in regulation of antigen presentation) was up-regulated only in spleen, while *cd2* (implied in adhesion T cell-APC through CD58 protein) was up-regulated only in head kidney. On the other hand, transcripts down-regulated in both organs were *cd8b* (T-cytotoxic cell marker), *cd79a* (associated with membrane-bound immunoglobulin in B-cells), membrane-bound form and long and short secretory forms of *cd80/86* (T cell activation), *cd163* (scavenger receptors of activated monocytes and macrophages), and *cd276* (costimulatory molecule of CD80/86). In the head kidney, down-regulation of *cd200* (NK immunosuppressive cell surface gene) was also observed.

Among the probes classified as macrophage-related genes, the genes up-regulated in both organs were *lectin1*, natural killer cell enhancement factor *nkef*, macrophage migration inhibitory factor *mif*, leucocyte elastase inhibitor *elast inh*, *plastin2*, a dendritic cell related protein, *ckin* (involved in cell adhesion), leucocyte cell derived chemotaxin-2 *lect2*, neutrophil cytosolic factors *ncf2* and *ncf4*, and *vcam* (vascular cell adhesion molecule or CD106) (Figure 5B). Up-regulated only in spleen were macrophage colony-stimulating factor precursor *mcsf pre* and receptor *mcsfr* and only in head kidney *mcsf1.2*, whereas *mcsfpre* was down-regulated in the head kidney. Down-regulated probes common to both organs were natural resistance-associated macrophage protein-alpha and -beta, bradykinin receptor B2 *kinr*, *clathrin*, and simple type II keratin *k8b*.

The cytokine inducible isoform of nitric oxide synthase (*inos*), which plays a role in the respiratory burst of leucocytes, was down-regulated in both organs.

#### Antimicrobial peptides, TLRs and complement

**Figure 6:**
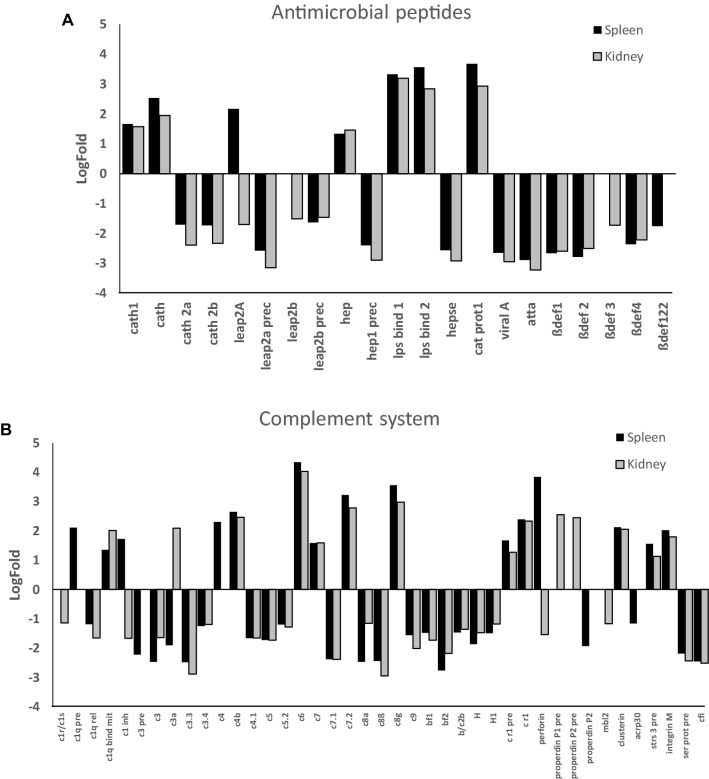
**Differential transcript expression of antimicrobial peptides (A) and complement system (B) in** ***L. garvieae*-infected trout.** Trout were injected with *L. garvieae* and RNA was extracted from spleen and head kidney of symptomatic (*n* = 6) and of control (*n* = 6) fish for microarray hybridization. After normalization, the mean and standard deviations were represented as differential expression LogFold from up- (+) or down- (−) regulated transcripts (> 1 or < −1, respectively), calculated by the formula, normalized fluorescence of each gene in *L. garvieae*-infected trout/normalized fluorescence of each gene in uninfected trout.

Several transcripts for antimicrobial peptides were up-regulated in the spleen and head kidney of *L. garvieae*-infected trout (Figure 6A). Among these, the transcripts for *cathelicidin 1*, *hepcidin*, LPS binding protein/bactericidal/permeability-increasing protein *lps bind 1* and *2*, and *cat prot1* (nonspecific cytotoxic cell cationic anti-microbial protein 1). Differential expression of liver-expressed antimicrobial peptide *leap2a* was found in both organs, with up-regulation in spleen and down-regulation in head kidney, while *leap2b* was down-regulated only in head kidney and no regulation was found in the spleen. On the other hand, several β-defensins, *cathelicidin 2a* and *2b*, *hepcidin 1 precursor*, *leap2a* and *2b precursors*, *hepse* (LPS heptosyltransferase), *viral A* (viral A-type inclusion protein repeat containing protein) and *atta* (antimicrobial peptide attacin AttA) were down-regulated in both spleen and head kidney of *L. garvieae*-infected trout (Figure 6A).

Some TLR transcripts were differentially expressed. These include up-regulation in the spleen of a *tlr* (AJ628348.1), that blasted to a recent GenBank entry for TLR22 (a fish-specific TLR), *tlr3* (recognizes viral dsRNA) and *tlr3 promotor*, *tlr13* (the ligand is bacterial RNA) and Toll-interacting protein (*tollipi*), whereas *tollipii* was up-regulated in the spleen and head kidney of *L. garvieae*-infected trout. By contrast, membrane and secreted form of TLR5 (recognizes bacterial flagellins), *tlr8a1* and *tlr8a2* (phagocytized bacterial RNA) were down-regulated in both organs. Up-regulation of TLR22 in the spleen and down-regulation of secreted TLR5 and TLR8a2 in spleen and head kidney of *L. garvieae*-infected fish were confirmed by RT-qPCR (Additional file 4). No significant differences were found in TLR1 (bacterial lipoprotein), TLR2 (Gram + bacteria, lipoteichoic acids and/or peptidoglycans) by RT-qPCR, confirming the microarray results.

In both spleen and head kidney of *L. garvieae*-infected trout, transcripts for the complement component c6 were among the top 50 up-regulated transcripts (Additional file 3). Other transcripts up-regulated in both organs were *c1q bind mit* (classical complement pathway); *c4b* (classical and lectin pathways); *c7*, *c7.2* and *c8* *g* (downstream membrane attack complex); *cr1*, *cr1 precursor* and *integrin M* (complement receptor system); and *clusterin* (complement regulation) (Figure 6B). In the spleen, *c1q precursor*, *c4*, and *c1 inhibitor* were also up-regulated. In the head kidney, *c3a* and *properdin p1*-*p2 precursors* (alternative pathway) appeared up-regulated. On the other hand, *c3*, *c3.3*, *c3.4*; *c1q rel* (classical pathway); *c4.1* (classical and lectin pathways); *bf1*, *bf2*, *b/c2b* (alternative pathway); *c5*, *c5.2*, *c7.1*, *c8a*, *c8b*, *c9* (downstream membrane attack complex); and *H*, *H1*, *cfi* (inhibitory factors) were down-regulated in both organs. In addition, *c3 precursor*, *properdin p2* and *acrp30* (complement related protein) were down-regulated only in spleen and *c1r/c1s* (classical pathway), and *mbl2* (lectin pathway) were down-regulated only in head kidney.

#### Cell signalling: chemokines and cytokines and their receptors

**Figure 7:**
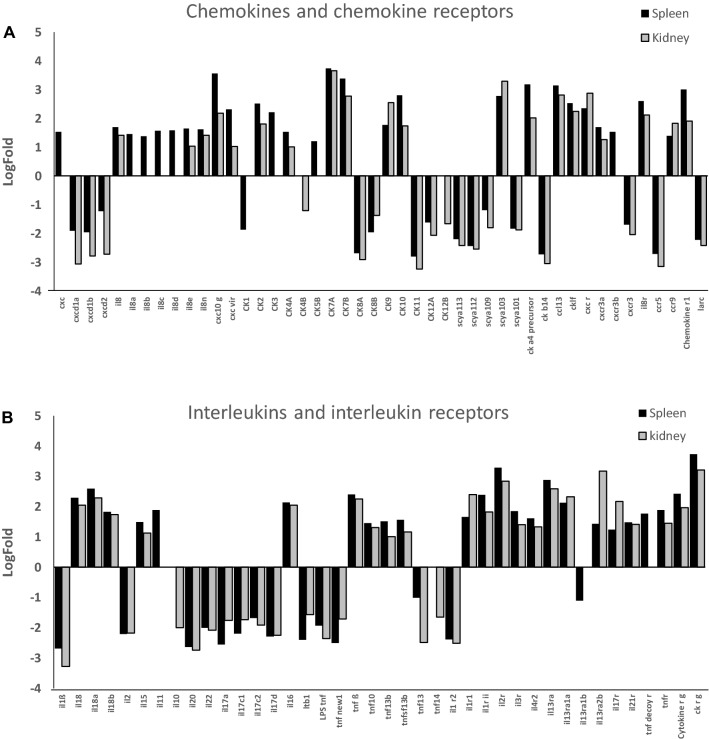
**Differential transcript expression of the chemokines and chemokine receptors (A) and interleukins and interleukin receptors (B) in** ***L. garvieae*-infected trout.** Trout were injected with *L. garvieae* and RNA was extracted from spleen and head kidney of symptomatic (*n* = 6) and of control (*n* = 6) fish for microarray hybridization. After normalization, the mean and standard deviations were represented as differential expression Log Fold from up- (+) or down- (−) regulated transcripts (> 1 or < −1, respectively), calculated by the formula, normalized fluorescence of each gene in *L. garvieae*-infected trout/normalized fluorescence of each gene in uninfected trout.

*cxcl10*-*like* was strongly up-regulated in the spleen and the head kidney of *L. garvieae*-infected trout (Figure 7A). Other CXC chemokines that were up-regulated to a lower extent in both organs included *cxcl8*, *cxcl8e and cxcl8n* (*il8*), and a virus-induced cxc chemokine (*cxcvir*). Other IL8 related probes including *il8a*, *il8b*, *il8c* and *il8d* were up-regulated in spleen. Regarding CC chemokines, *ck7a* and *ck7b* were strongly up-regulated in both organs, together with *ck2*, *ck4a*, *ck9*, *ck10*, small inducible cytokine *scya 103*, *ccl13*, *cka4 precursor* and a *chemokine*-*like factor*. *ck3* and *ck5b* were also up-regulated in the spleen. As for chemokine receptors, *cxcr3a*, *il8r*, ccr9 and a *chemokine receptor 1* appeared up-regulated in both organs, and *cxcr3b* also in the spleen (Figure 7A). On the other hand, down-regulated transcript for *ck1* was found in spleen, and for *ck4b* and *ck12b* in head kidney, whereas other chemokines and chemokine receptors were down-regulated in both organs (Figure 7A).

Regarding interleukins and their receptors, up-regulated probes in both organs are shown in Figure 7B. However, *tnf*-*decoy* receptor was only up-regulated in spleen. Cytokines such as *il10* and *tnf14 were d*own-regulated only in the head kidney. Other cytokines, such as the pro-inflammatory *il1b*, were strongly down-regulated in both organs (Figure 7B).

Multiple probes for type 1 IFN 2, 3, 4 and 5, and for IFN gamma appeared down-regulated in the spleen and the head kidney of *L. garvieae*-infected trout. On the contrary, probes for interferon regulatory factors *irf1*, *irf2*, *stat1* (signal transducer and activator factor, involved in IFN signalling pathways), and several interferon inducible genes were up-regulated in both organs, including the mixovirus resistance GTPase genes Mx (Additional file 5) or *ifn induced proteins 1*, *2*, *gig2*, *30*, *35kd*.

In addition, many viruses induced genes were up-regulated in both organs following *L. garvieae* infection, including *vigs 1*, *2*, *3*, *4*, *5*, *6*, *7*, *9*, (*vig10* only in spleen), *b32*, *b51*, *b143*, *b191*, *b203*, *b225*, (*b124* only in spleen) (Additional file 5).

The suppressors of cytokine signalling *socs1*, *socs3*, *socs5 and socs7* were up-regulated in the spleen and head kidney, whereas *socs2* was up-regulated only in the head kidney.

#### Apoptosis, heat shock proteins and transcription factors

Regarding the genes involved in the apoptotic process, *apoptosis regulator BAX membrane isoform*, caspase transcripts for *casp9* (initiator caspase) and *annexin a1* were up-regulated in both organs of *L. garvieae*-infected fish.

Many transcripts grouped as heat shock proteins were strongly up-regulated in *L. garvieae*-infected fish, including probes for *hsp90* (chaperone with roles in stabilization and folding of proteins and protein degradation), transcripts for genes with roles in protein degradation as *collagenase*, *chitinase*, *proteasome subunit9* (*lmp2*) and *ubiquitin*, different probes for several *cathepsins*, *prefoldin and prostaglandin e synthase3* (Additional file 6).

Furthermore, up to 461 and 443 transcription factors were modulated in the spleen and head kidney, respectively. Among them, is remarkable the up-regulation of key factors involved in the immune response such as *ap1*, *jun* (a proto-oncogen), *ccaat/enhancer binding protein alpha* and *delta* (CEBP), *nod3*, *p100/p52 (nfkb)* and *nuclear factor kappa B inhibitor alpha* in both organs; *stat5* and *mda5* in spleen, *b*-*cell translocation gene3* in head kidney. Strong up-regulation of transcription factor 3 *btf3* (required for transcriptional initiation), of *cyclin B1* and *cyclin B2* was observed in both organs. Down-regulation of *tdt* (terminal deoxynucleotidyl transferase, participates in antibody gene recombination) in both organs, and *pax5* (expressed at early but not late stages of B-cell differentiation) in head kidney was also observed.

## Discussion

In this work, we have performed a transcriptome analysis to study the immune responses in the spleen and head kidney of trout with clinical signs of lactococcosis 72 h after *L. garvieae* inoculation, to analyze a fully developed response. Despite the clinical and economical relevance of *L. garvieae* as a fish pathogen for the worldwide rainbow trout farm industry [1, 2], studies on the immune response against *L. garvieae* are lacking in this fish species. Therefore, this study will contribute to a better understanding of the host–pathogen interaction during *L. garvieae* infections.

Most studies that investigated the immune transcriptome during bacterial infections in fish have used microarrays designed from EST derived from whole genomes in which transcripts of many immune-related genes are often under-represented [12]. To reduce this drawback, we used a custom designed immune-targeted microarray specifically containing transcripts derived from immune-related mRNAs deposited in public gene or pathway data banks [13]. As a result, the 15KID032303 custom designed microarray used in this work contains approximately threefold more unique immune-related probes than other microarrays used in similar fish studies [24]. The present microarray has already been successfully used to study the immune response to different pathogens and/or vaccination in rainbow trout [13–16].

The obtained results showed a similar transcriptome profile for the immune response between the spleen and the head kidney in *L. garvieae*-infected trout 72 h post- inoculation, a time at which the infected trout exhibited external and internal signs of lactococcosis. These results are in accordance with those reported in the earlier immune response to *L. garvieae* in grey mullet (*Mugil cephalus*), where similar immune responses were found in spleen and head kidney [11] despite the different post-inoculation times (24 h in the grey mullet *versus* 72 h in rainbow trout). Thus, Byadgi et al. [11] analyzed the early immune response to *L. garvieae* infection before development of lactococcosis, while we analyzed a later immune response after fish exhibited obvious signs of lactococcosis in this study. Early immune responses are fundamentally important, as they provide the first line of defense against the infection, while we focused our study at 72 h post-injection, when the immune response is more developed.

The most important difference observed between spleen and head kidney was the high inflammatory response detected by histopathology and immunohistochemistry in the spleen corresponding to granulomatous lesions containing macrophages with internalized *L. garvieae* (Figure 2B). These results are consistent with splenomegaly, commonly observed as one of the most important clinical signs of lactococcosis [1, 2, 25]. Granuloma formation could be easily correlated with the up-regulation of the chemokines CXCL10, CXCL8 (also called IL8) and their receptors (CXCR3 and IL8R respectively) and other inflammatory cytokines and macrophage marker genes in the spleen (Figures 5B and 7), and the increase in transcription of genes related also to neutrophils in this tissue (Table 1). In trout, IL8 plays a key role in the recruitment of monocytes and neutrophils into tissues [26] and in the activation of phagocytosis in macrophages. Activated macrophages are key to further activate NK cells, cytotoxic T cells, Th1 and Th2 lymphocytes and macrophages, amplifying the inflammatory response in the granulomas. Phagocytosis is also a key function in fish B cells [27, 28]. Phagocytosis is known to be triggered by the recognition of pathogen-associated molecular patterns (PAMPs) with receptors in the phagocytes (such as complement receptors, Toll receptors and NOD-like receptors). This recognition promotes phagocytosis and the induction of pro-inflammatory cytokines that are involved in initiation of the adaptive immune processes.

TLR5 is usually up-regulated in response to flagellin stimulation. In *L. garvieae* infected samples, TLR5 was down-regulated at low fold changes (Additional file 4). This result was not unexpected, since *L. garvieae* is not flagellated. On the other hand, TLR5 has been found either up- or down-regulated in tissues of infected fish, regardless the presence of flagellin in the microorganisms [15, 29].

By contrast, TLR22, TLR13 (bacterial RNA) and NOD-linked signalling pathways, including NOD and MAPK, were up-regulated in the spleen (Table 2). According to the KEGG database, these gene pattern up-regulations in mammals could be related to the intracellular recognition of bacterial peptidoglycans, lipoteichoic acids and muramyl dipeptide in the phagosome, suggesting that these antigens may act as inducers for fish host responses against *L. garvieae* infection. The binding of these PAMPS to NOD-like receptors activates the transcription of NF-KB and triggers the production of antimicrobial peptides (AMPs) and other pro-inflammatory molecules, Although TLR22 has been associated with the recognition of long dsRNA mainly from viruses [30], TLR22 up-regulation has also been reported in the spleen in common carp and turbot after *Aeromonas hydrophila* and *Streptococcus iniae* infections, respectively [31, 32]. Recent studies indicate that TLR22 can be modulated by PAMPS present in bacteria, such as peptidoglycans and lipopolysaccharides [32–34]. Therefore, TLR22 may be considered as an important pathogen surveillance receptor able to link the innate and adaptative immune pathways. Under this view, up-regulation of TLR22 in the spleen after *L. garvieae* infection could initiate the activation of adaptive immune response. This is in line with the increase of melanomacrophages in the spleen (Figure 1A, B). Studies, across many fish species, identified the melanomacrophage centers as primitive functional germinal centers involved in the adaptive immune response activation [35]. The up-regulation of secreted IgM in the spleen and head kidney of *L. garvieae*-infected trout, confirmed by RT-qPCR (Figure 4), as well as the up-regulation of BT cells only in the spleen (Table 2) could indicate the further development of an antibody response by both IgM (+) and IgT (+) spleen B cells to respond to systemic infection. In trout, IgT is involved in mucosal immunity and it also participates in systemic immunity [36, 37].

Different chemokines and interleukins were up-regulated in the trout spleen after *L. garvieae* infection. Among these, CK5B and IL11 have been reported as greatly induced by LPS and bacteria [38, 39]. Despite IL1-β is an important mediator of early infection responses and an important cytokine linked to inflammation [40], its down-regulation after 72 h of infection, as it is shown in this work, could indicate decrease in the innate inflammatory responses at this point. The same situation has been observed respect to the cytokine inducible isoform of nitric oxide synthase (*inos*), which was down-regulated in spleen and head kidney. This is also correlated with the down-regulation of C3 (and factor *H1* also in the spleen), but with the up-regulation of final C6 component of complement pathways, and the induction of regulatory T cells in both tissues. In addition, several genes for protein degradation and apoptosis are up-regulated in both organs that correlate with a late phase of the inflammatory process.

We have observed a low down-regulation of IFN type I and IFN gamma genes in the tissues of *L. garvieae* infected fish but several interferons stimulated genes (ISG) appeared up-regulated (Additional file 5). This could be correlated with the intracellular location of the bacteria observed previously both in vitro and in vivo [19]. Although Interferon type I responsive genes are not usually induced by bacterial infections, ISG have been also observed up-regulated after *Aeromonas salmonicida* challenge in trout early stages [41].

In addition to the induction of macrophages and neutrophils in the spleen, the positive regulation of MHC I molecules, cytotoxic T cells and NK cells in the kidney suggest a potential intracellular mode of pathogenicity for this bacterium, which is in line with the ability of *L. garvieae* to invade non-phagocytic cells [19] and the formation of granulomas. Moreover, the up-regulation of MHC I was also observed in grey mullets infected with *L. garvieae* [11], proposing this possibility.

Overall, our results show that *L. garvieae* infection induced a robust inflammatory response in the trout spleen, which correlated with the increase of melanomacrophages and presence of granulomas in this tissue. A correlation also existed between the presence of granulomas and up-regulated gene sets in the spleen, which defined inflammatory molecules, macrophages and neutrophils, whereas NK cells, cytotoxic cells, and equivalents to regulatory T cells were up-regulated in head kidney. In the spleen, there is also an activation of adaptive immune mechanisms and the development of an antibody response by both IgM (+) and IgT (+) spleen B cells to respond to systemic infection against *L. garvieae*. This study can aid in the design of new vaccination strategies to prevent the effects of this disease in trout.

## Additional files

**Additional file 1.** **Schematic representation of the array distribution.**

**Additional file 2.** **Primers used for expression analysis by RT-qPCR.**

**Additional file 3.** **Top 50 up- and down-regulated probes in spleen and head kidney.** Differentially top 5o regulated probes are marked in bold.

**Additional file 4.** **Correlation between RT-qPCR and microarray data.** The log_10_ values of the RT-qPCR expression levels in spleen (S) or kidney (K) of infected fish were plotted against the microarray log_10_ values for six representative genes.

**Additional file 5.** **Differential transcript expression of Mx genes and virus induced genes (vig).** Trout were injected with *L. garvieae* and RNA was extracted from spleen and head kidney of symptomatic (*n* = 6) and of control (*n* = 6) fish for microarray hybridization. After normalization, the mean and standard deviations were represented as differential expression Log Fold from up- (+) or down- (−) regulated transcripts (> 1 or < −1, respectively), calculated by the formula, normalized fluorescence of each gene in *L. garvieae*-infected trout/normalized fluorescence of each gene in uninfected trout.

**Additional file 6.** **Differential transcript expression of heat shock proteins.** Trout were injected with *L. garvieae* and RNA was extracted from spleen and head kidney of symptomatic (*n* = 6) and of control (*n* = 6) fish for microarray hybridization. After normalization, the mean and standard deviations were represented as differential expression Log Fold from up- (+) or down- (−) regulated transcripts (> 1 or < −1, respectively), calculated by the formula, normalized fluorescence of each gene in *L. garvieae*-infected trout/normalized fluorescence of each gene in uninfected trout.
