# Supplementary material for: Spleen and head kidney differential gene expression patterns in trout infected with Lactococcus garvieae correlate with spleen granulomas
Source: Vet Res. 2019 May 2;50:32. doi: 10.1186/s13567-019-0649-8 (PMC6498643; doi:10.1186/s13567-019-0649-8)
Supplement: Supplementary file 1 — Additional file 1. Schematic representation of the array distribution. [file 13567_2019_649_MOESM1_ESM.pptx]

## Slide 1
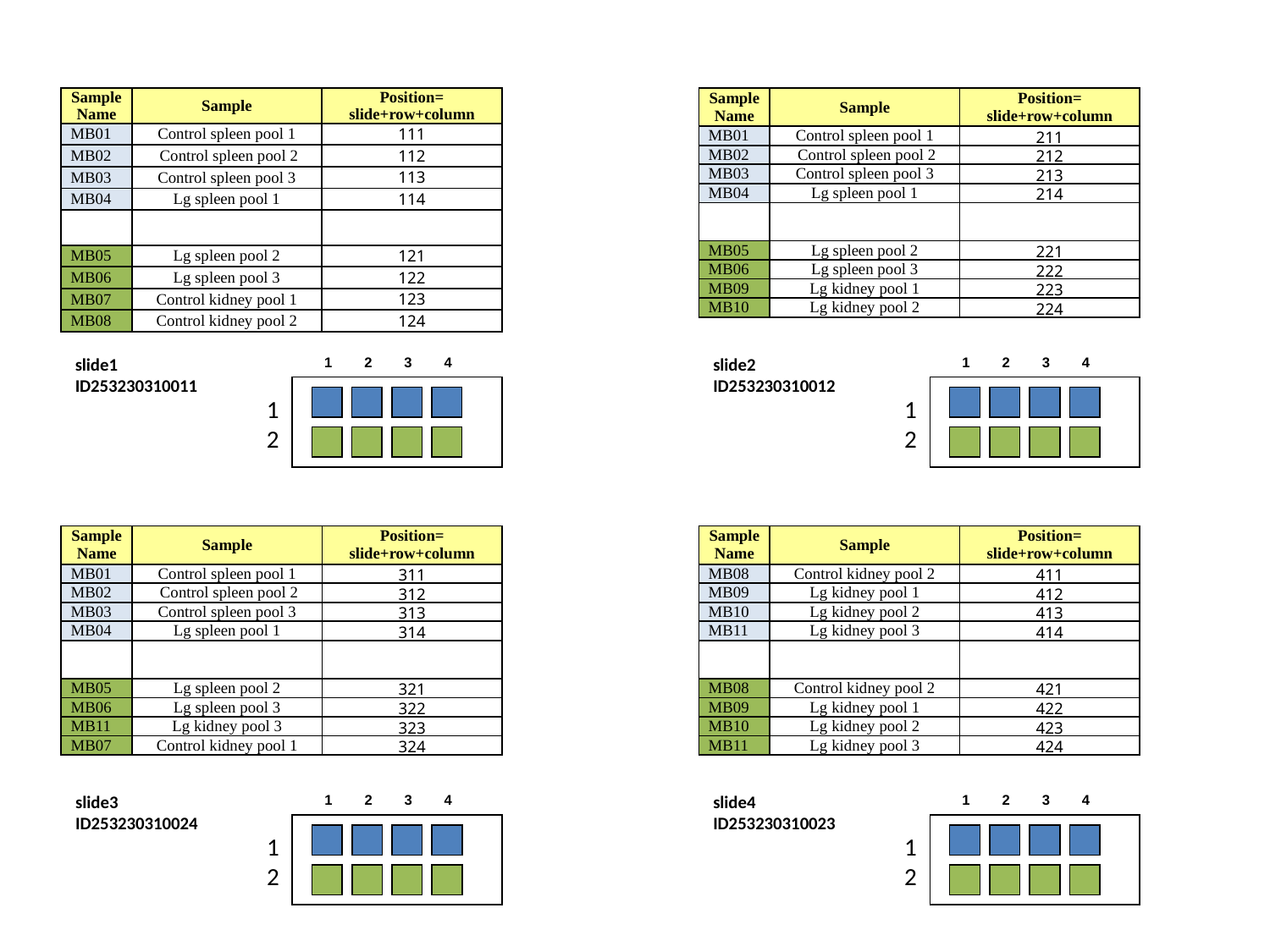

| Sample Name | Sample | Position= slide+row+column |
| --- | --- | --- |
| MB01 | Control spleen pool 1 | 111 |
| MB02 | Control spleen pool 2 | 112 |
| MB03 | Control spleen pool 3 | 113 |
| MB04 | Lg spleen pool 1 | 114 |
| | | |
| MB05 | Lg spleen pool 2 | 121 |
| MB06 | Lg spleen pool 3 | 122 |
| MB07 | Control kidney pool 1 | 123 |
| MB08 | Control kidney pool 2 | 124 |
| Sample Name | Sample | Position= slide+row+column |
| --- | --- | --- |
| MB01 | Control spleen pool 1 | 211 |
| MB02 | Control spleen pool 2 | 212 |
| MB03 | Control spleen pool 3 | 213 |
| MB04 | Lg spleen pool 1 | 214 |
| | | |
| MB05 | Lg spleen pool 2 | 221 |
| MB06 | Lg spleen pool 3 | 222 |
| MB09 | Lg kidney pool 1 | 223 |
| MB10 | Lg kidney pool 2 | 224 |
slide1
ID253230310011
slide2
ID253230310012
1 2 3 4
1 2 3 4
1
2
1
2
| Sample Name | Sample | Position= slide+row+column |
| --- | --- | --- |
| MB01 | Control spleen pool 1 | 311 |
| MB02 | Control spleen pool 2 | 312 |
| MB03 | Control spleen pool 3 | 313 |
| MB04 | Lg spleen pool 1 | 314 |
| | | |
| MB05 | Lg spleen pool 2 | 321 |
| MB06 | Lg spleen pool 3 | 322 |
| MB11 | Lg kidney pool 3 | 323 |
| MB07 | Control kidney pool 1 | 324 |
| Sample Name | Sample | Position= slide+row+column |
| --- | --- | --- |
| MB08 | Control kidney pool 2 | 411 |
| MB09 | Lg kidney pool 1 | 412 |
| MB10 | Lg kidney pool 2 | 413 |
| MB11 | Lg kidney pool 3 | 414 |
| | | |
| MB08 | Control kidney pool 2 | 421 |
| MB09 | Lg kidney pool 1 | 422 |
| MB10 | Lg kidney pool 2 | 423 |
| MB11 | Lg kidney pool 3 | 424 |
slide3
ID253230310024
slide4
ID253230310023
1 2 3 4
1 2 3 4
1
2
1
2
