# Supplementary material for: Spleen and head kidney differential gene expression patterns in trout infected with Lactococcus garvieae correlate with spleen granulomas
Source: Vet Res. 2019 May 2;50:32. doi: 10.1186/s13567-019-0649-8 (PMC6498643; doi:10.1186/s13567-019-0649-8)
Supplement: Supplementary file 2 — Additional file 2. Primers used for expression analysis by RT-qPCR. [file 13567_2019_649_MOESM2_ESM.docx]

**Additional file 2. Primers used for expression analysis by real-time PCR.**

| **Gene** | **Forward sequence 5’-3’** | **Reverse sequence 5’-3’** | **Reference** |
| --- | --- | --- | --- |
| EF-1α* | CAAGGATATCCGTCGTGGCA | ACAGCGAAACGACCAAGAGG | [22] |
| IgM secreted | TACAAGAGGGAGACCGGAGGAGT | CTTCCTGATTGAATCTGGCTAGTGGT | [22] |
| Cathelicidin (Cath 1) | ACCAGCTCCAAGTCAAGACTTTGAA | TGTCCGAATCTTCTGCTGCAA | [22] |
| TLR-1 | CAGACGCCCTGTTGATGTTC | CCTTCACAAGTTCCACCACG | [23] |
| TLR-2 | GATCCAGAGCAACACTCTCAACAT | CTCCAGACCATGAAGTTGACAAAC | [23] |
| TLR-5 membrane | GCGCATCACTTCAGGGGGAT | GCATTTCACCACTTGCAGGTAGA | [22] |
| TLR-5 secreted | GCGCTCATAACTTCAGGGGGAT | GCATTTCACCACCTGCAGGTATT | [22] |
| TLR-8a2 | CATCTATGTTCTCATCCAGCAACC | GGTCCCCCTAATAGACAACCTCTT | [23] |
| TLR-22 | TGGACAATGACGCTCTTTTACC | GAGCTGATGGTTGCAATGAGG | [23] |

*House-keeping gene.
