# Supplementary figures and images for: Spleen and head kidney differential gene expression patterns in trout infected with Lactococcus garvieae correlate with spleen granulomas
Source: Vet Res. 2019 May 2;50:32. doi: 10.1186/s13567-019-0649-8 (PMC6498643; doi:10.1186/s13567-019-0649-8)

## Slide 1
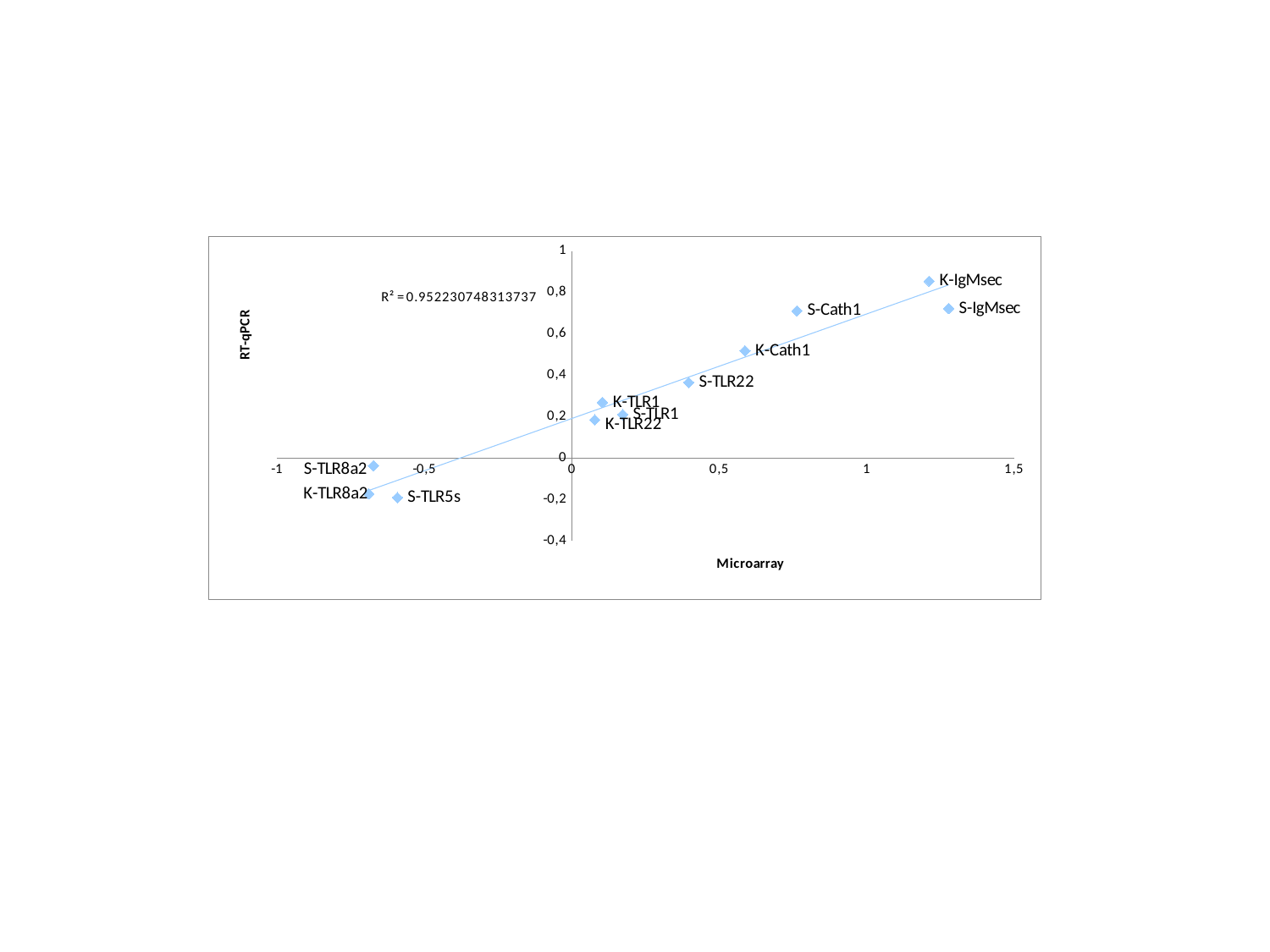

### Chart
| Category | S-Cath1 |
|---|---|

Supplement: Supplementary file 4 — Additional file 4. Correlation between RT-qPCR and microarray data. The log10 values of the RT-qPCR expression levels in spleen (S) or kidney (K) of infected fish were plotted against the microarray log10 values for six representative genes. [file 13567_2019_649_MOESM4_ESM.pptx]
