# Supplementary material for: Spleen and head kidney differential gene expression patterns in trout infected with Lactococcus garvieae correlate with spleen granulomas
Source: Vet Res. 2019 May 2;50:32. doi: 10.1186/s13567-019-0649-8 (PMC6498643; doi:10.1186/s13567-019-0649-8)
Supplement: Supplementary file 5 — Additional file 5. Differential transcript expression of Mx genes and virus induced genes (vig). Trout were injected with L. garvieae and RNA was extracted from spleen and head kidney of symptomatic (n = 6) and of control (n = 6) fish for microarray hybridization. After normalization, the mean and standard deviations were represented as differential expression Log Fold from up- (+) or down- (−) regulated transcripts (> 1 or < −1, respectively), calculated by the formula, normalized fluorescence of each gene in L. garvieae-infected trout/normalized fluorescence of each gene in uninfected trout. [file 13567_2019_649_MOESM5_ESM.pptx]

## Slide 1
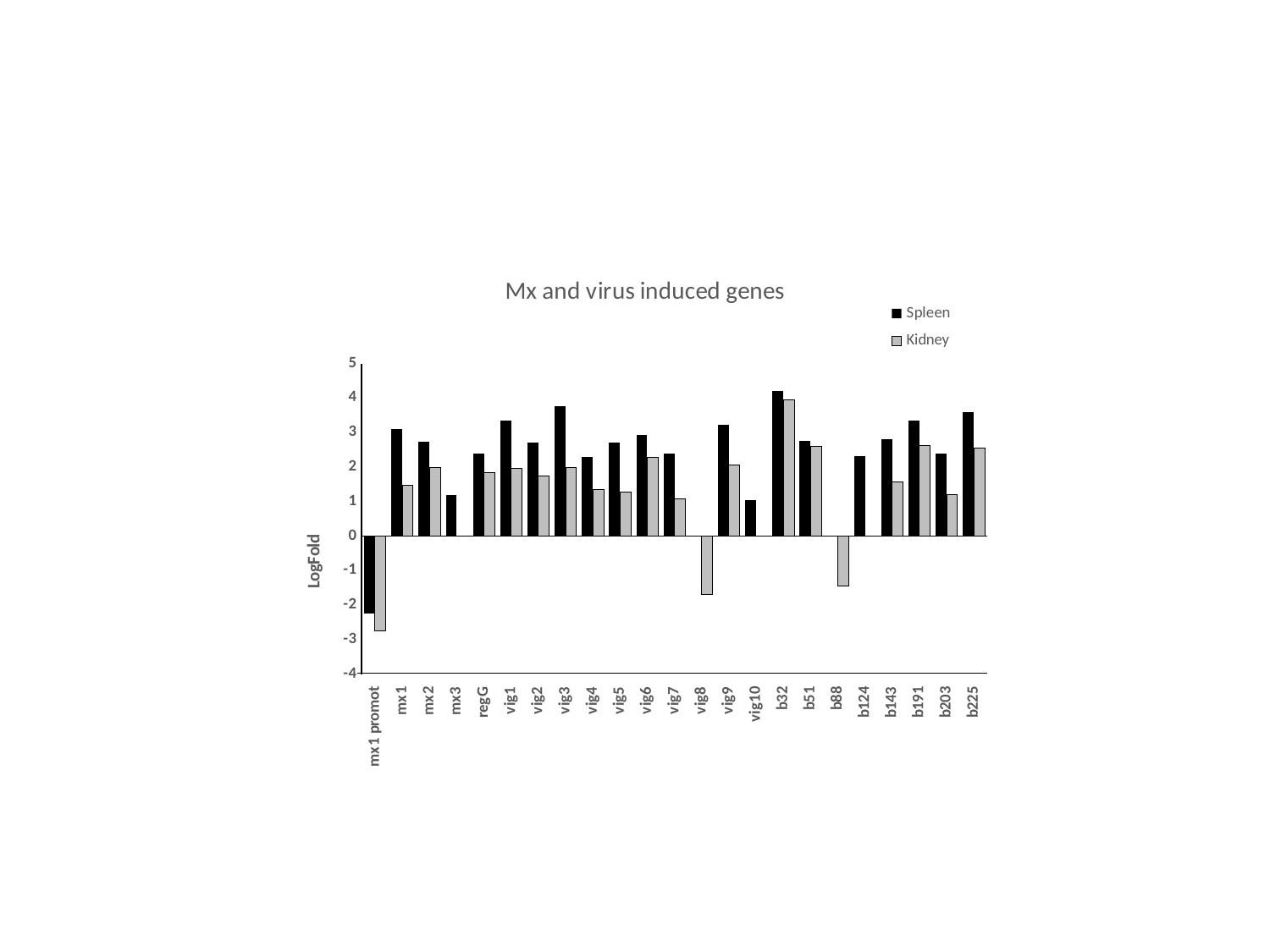

### Chart: Mx and virus induced genes
| Category | | |
|---|---|---|
| mx1 promot | -2.25262719406339 | -2.74093174914947 |
| mx1 | 3.09085259622827 | 1.47475499798484 |
| mx2 | 2.73552634661073 | 1.97713060754176 |
| mx3 | 1.18130779555967 | None |
| regG | 2.39923511727842 | 1.84620263771428 |
| vig1 | 3.34437237179428 | 1.96558380853678 |
| vig2 | 2.71289132704315 | 1.7283144099729 |
| vig3 | 3.76356367510187 | 1.98957824522372 |
| vig4 | 2.28198803344235 | 1.34013488024186 |
| vig5 | 2.70514583926679 | 1.28064729091965 |
| vig6 | 2.94055967202341 | 2.27741147419116 |
| vig7 | 2.37906290502577 | 1.08722012680927 |
| vig8 | None | -1.70234668251543 |
| vig9 | 3.2239878391281 | 2.04964630385764 |
| vig10 | 1.03133442601239 | None |
| b32 | 4.21034906957776 | 3.94396073030232 |
| b51 | 2.76914469978658 | 2.59874623951865 |
| b88 | None | -1.45039610175105 |
| b124 | 2.31625826447752 | None |
| b143 | 2.80127940396872 | 1.56867644019449 |
| b191 | 3.34847231821787 | 2.62048668009287 |
| b203 | 2.38965455674645 | 1.19524045069729 |
| b225 | 3.59928914890775 | 2.53857918729102 |
