# Supplementary material for: Spleen and head kidney differential gene expression patterns in trout infected with Lactococcus garvieae correlate with spleen granulomas
Source: Vet Res. 2019 May 2;50:32. doi: 10.1186/s13567-019-0649-8 (PMC6498643; doi:10.1186/s13567-019-0649-8)
Supplement: Supplementary file 6 — Additional file 6. Differential transcript expression of heat shock proteins. Trout were injected with L. garvieae and RNA was extracted from spleen and head kidney of symptomatic (n = 6) and of control (n = 6) fish for microarray hybridization. After normalization, the mean and standard deviations were represented as differential expression Log Fold from up- (+) or down- (−) regulated transcripts (> 1 or < −1, respectively), calculated by the formula, normalized fluorescence of each gene in L. garvieae-infected trout/normalized fluorescence of each gene in uninfected trout. [file 13567_2019_649_MOESM6_ESM.pptx]

## Slide 1
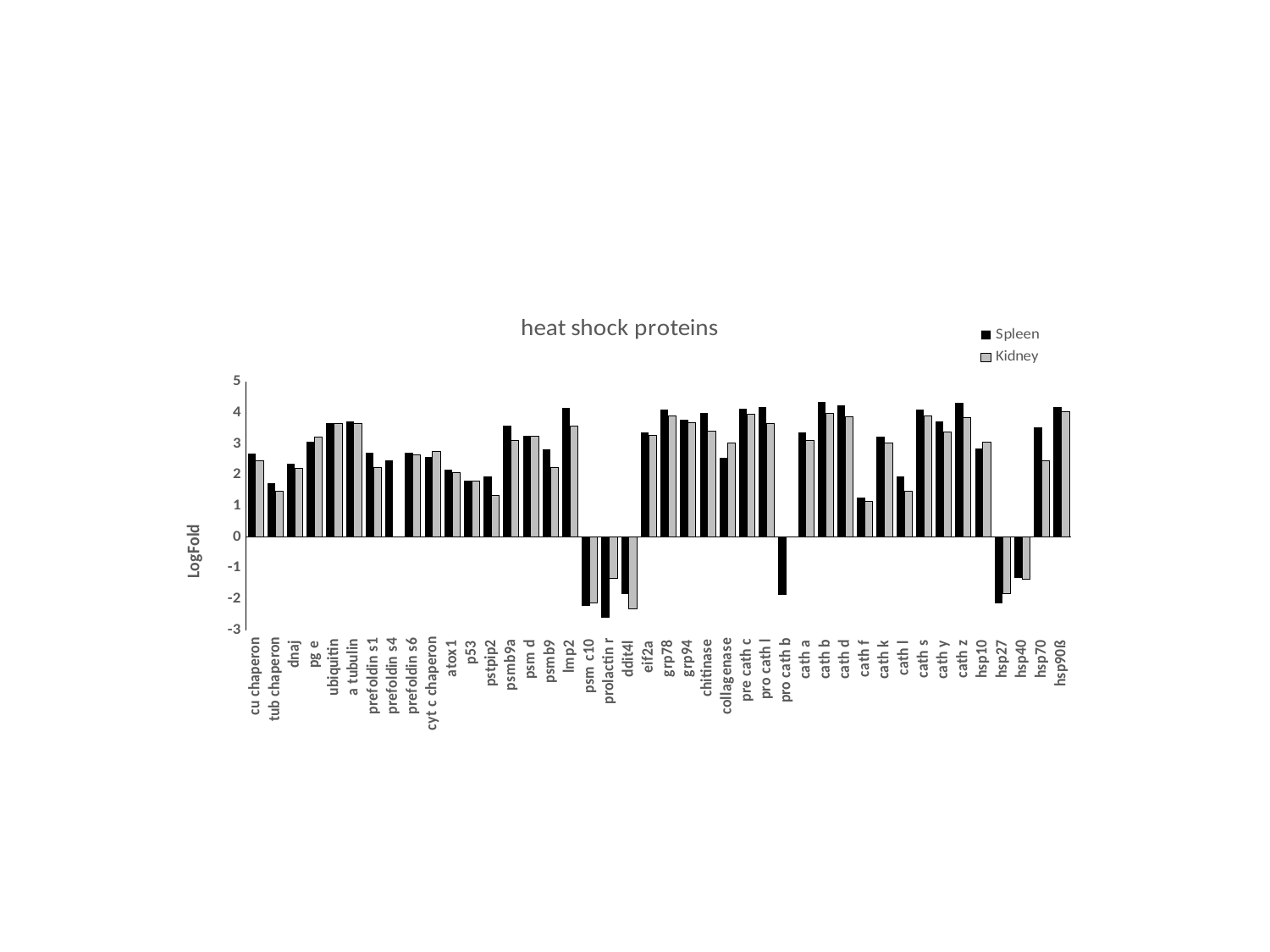

### Chart: heat shock proteins
| Category | | |
|---|---|---|
| cu chaperon | 2.70322263104308 | 2.46460852215381 |
| tub chaperon | 1.73032753232731 | 1.47931235930136 |
| dnaj | 2.36702837271654 | 2.21837126216951 |
| pg e | 3.06879563232859 | 3.22810012944189 |
| ubiquitin | 3.67543049931007 | 3.6471336372415 |
| a tubulin | 3.71808155550403 | 3.66843101483969 |
| prefoldin s1 | 2.71781117199438 | 2.25057764778217 |
| prefoldin s4 | 2.46452402452467 | None |
| prefoldin s6 | 2.71781117199438 | 2.65347880292483 |
| cyt c chaperon | 2.59054140001775 | 2.76527980175474 |
| atox1 | 2.1654797044377 | 2.06409998769886 |
| p53 | 1.81036667076761 | 1.79703292917807 |
| pstpip2 | 1.9661699101491 | 1.33570442926762 |
| psmb9a | 3.60164828626575 | 3.10171849870527 |
| psm d | 3.26324461237035 | 3.25696172063272 |
| psmb9 | 2.83667376147291 | 2.23731519654143 |
| lmp2 | 4.16064421939399 | 3.56861634412467 |
| psm c10 | -2.20905333188108 | -2.11525807625174 |
| prolactin r | -2.58981493195637 | -1.34279457426056 |
| ddit4l | -1.82759636050621 | -2.31421994136095 |
| eif2a | 3.38420741849269 | 3.28845470736156 |
| grp78 | 4.10806393710551 | 3.9093296509309 |
| grp94 | 3.76698784164311 | 3.68926722133354 |
| chitinase | 4.01069093136763 | 3.42509180008007 |
| collagenase | 2.54027076341954 | 3.04031079213854 |
| pre cath c | 4.13690918809849 | 3.94569179120701 |
| pro cath l | 4.18169112819449 | 3.66401235454172 |
| pro cath b | -1.85623442995938 | None |
| cath a | 3.37448042027646 | 3.1012453996435 |
| cath b | 4.3447323504368 | 3.97701389202842 |
| cath d | 4.23746974039446 | 3.87952771268165 |
| cath f | 1.25944849663404 | 1.14602135443053 |
| cath k | 3.22413973854407 | 3.02087519187464 |
| cath l | 1.96622432933528 | 1.46965793066311 |
| cath s | 4.09976204447377 | 3.89279580902712 |
| cath y | 3.73077541207443 | 3.39003589383335 |
| cath z | 4.33747394695694 | 3.83742072136578 |
| hsp10 | 2.84453553469394 | 3.05388197326107 |
| hsp27 | -2.13421524783078 | -1.82978778291157 |
| hsp40 | -1.30654860389054 | -1.37007585337431 |
| hsp70 | 3.53986179097821 | 2.47253468400158 |
| hsp90ß | 4.19592110608072 | 4.05318510424463 |
